# Let Them Eat Fish!—Exploring the Possibility of Utilising Unwanted Catch in Food Bank Parcels in The Netherlands

**DOI:** 10.3390/foods10112775

**Published:** 2021-11-11

**Authors:** Madhura Rao, Lea Bilić, Joanna Duwel, Charlotte Herentrey, Essi Lehtinen, Malin Lee, María Alejandra Díaz Calixto, Aalt Bast, Alie de Boer

**Affiliations:** 1 Campus Venlo, Food Claims Centre Venlo, Faculty of Science and Engineering, Maastricht University, 5900 AA Venlo, The Netherlands; a.deboer@maastrichtuniversity.nl; 2 Campus Venlo, University College Venlo, Maastricht University, 5900 AA Venlo, The Netherlands; l.bilic@student.maastrichtuniversity.nl (L.B.); j.duwel@student.maastrichtuniversity.nl (J.D.); c.herentrey@student.maastrichtuniversity.nl (C.H.); e.lehtinen@student.maastrichtuniversity.nl (E.L.); malin.lee@student.maastrichtuniversity.nl (M.L.); m.diazcalixto@student.maastrichtuniversity.nl (M.A.D.C.); a.bast@maastrichtuniversity.nl (A.B.); 3 Department of Pharmacology & Toxicology, Faculty of Health, Medicine and Life Sciences, Maastricht University, 6229 ER Maastricht, The Netherlands

**Keywords:** landing obligation, Common Fisheries Policy, food waste, food security, sustainability

## Abstract

The Common Fisheries Policy of the European Union was reformed in 2013 with the aim of improving the sustainability of the fishing sector. The Landing Obligation, a cornerstone of this reform, requires fishers to land their unwanted catch instead of discarding it at sea. Existing literature pays little attention to what becomes of this unwanted catch once it is landed. To further the discourse on the sustainable valorisation of unwanted catch, this study explores whether unwanted catch that is safe for human consumption could be used for improving food security. The paper focuses on Dutch food banks, which deliver critical food aid to over 160,000 individuals yearly but struggle to provide all dependant recipients with nutritionally balanced food parcels. The research question is addressed in two ways. The food bank recipients’ willingness to consume UWC is evaluated quantitatively through a survey. Next to this, data from interviews with relevant stakeholders are analysed qualitatively. Results indicate that the Food Bank Foundation and its recipients are willing to receive this fish if it is safe to consume and accessible. However, various factors such as existing infrastructure, lack of economic incentive to donate, competition from non-food and black markets, and the fishing industry’s conflict with the landing obligation might pose barriers to this kind of valorisation. The dissonance between fisheries, food, and sustainability policies is discussed and identified as a key limiting factor. To bridge the differences between these policy areas, we propose public-private partnerships and voluntary agreements among involved stakeholders.

## 1. Introduction

As part of the 2013 Common Fisheries Policy (CFP) reform, the European Commission introduced a Landing Obligation (LO) which requires all catches of species subject to catch quotas and minimum conservation reference size to be landed and counted against quota. The objective of this requirement is to promote selective fishing and significantly reduce the incidence of unwanted catches (UWC). Despite implementing selective fishing strategies such as the use of specialised gear and locating species-specific hotspots, some untargeted fish are still likely to end up being caught and landed [1]. Considering that fishers have traditionally discarded such fish at sea, routes to fully utilise the landed UWC are currently underdeveloped. The 2015–2019 implementation period for the LO saw the sector focus on negotiating requirements and engineering ways to fish more selectively. However, little attention was paid to developing sustainable ways to utilise UWC that would be landed as a consequence of the LO.

Next to conserving marine biological resources, reducing waste was a key incentive in bringing about this reform [2]. When UWC with low survival rate is discarded at sea, it contributes neither to the health of the fish stock nor towards meeting the dietary needs of the growing population. Therefore, the practice of discarding UWC at sea can be seen as wasteful and undesirable [2]. However, not utilising this UWC once it is landed is as wasteful and undesirable, if not more. Given that meeting current and future protein needs of the population is a significant challenge for the food system [3], not making appropriate use of the landed UWC to supplement the human diet when feasible is a missed opportunity. This is true also for developed countries like the Netherlands, where over 1 million people out of the 17.4 million population are known to be part of low-income households [4]. Of these, several individuals require social assistance to procure sufficient food [4]. The national Food Bank Foundation, Voedselbanken Nederland, aids these individuals by providing weekly food parcels through its network of 171 food banks [5]. Based on existing literature, it is known that Dutch food banks, like their counterparts around the world, struggle to provide recipients with nutritionally balanced parcels [6,7]. In the Netherlands, fish and fruit are particularly scarce in food bank parcels [7]. Neter et al. (2018) found that 99% of the food bank recipients did not consume fish in amounts considered sufficient by the national dietary guidelines [7]. This issue cannot be ignored, as the number of food bank recipients continues to grow [5]. Although it is known that there is a lack of fish in food bank parcels, no previous studies have enquired as to whether food bank recipients are keen on consuming more fish if it is made available to them. Our study provides a first foray into this topic.

Together, the issues of UWC underutilisation and fish deficiency in food bank parcels concern several of the United Nations Sustainable Development Goals (UN SDGs), such as goal 2-zero hunger, goal 3-good health and well-being, goal 12-responsible consumption and production, and goal 14-life below water. The goals stand for eliminating hunger and food insecurity, ensuring a healthy life, making consumption and production more sustainable, and ensuring the sustainable use of marine resources. The SDGs give UN Member States, including the Netherlands, a blueprint for a more sustainable future. Although many actions have already been taken, most Member States are not on track to reach the goals by 2030 [8]. By sustainably utilising UWC and increasing the protein content of food bank parcels, the Netherlands would come closer to realising the SDGs. However, with existing EU policies, market conditions, and stakeholder relations, addressing the issue is not straightforward, and may require several large-scale changes.

Currently under the LO, UWC that is under the minimum conservation reference size (MRCS) cannot be sold for direct human consumption in the EU. Instead, it ends up as pet food, fish meal, or other products. According to Cashion et al. (2017), on a global scale as much as 90% of fish which are safe for human consumption end up being used for fishmeal, fish oil, and other non-food applications [9]. This does not align with the food waste hierarchy developed by Papargyropoulou et al. (2014), which states that before recycling food into animal feed, redistribution for human consumption must be considered, if feasible [10]. Iñarra et al. (2019) have adapted the hierarchy specifically for fish waste management, starting again with (1) prevention and reduction, and continuing to (2) human consumption, (3) bio-products, (4) animal feed, (5) industrial uses, (6) production of energy, (7) agronomic purposes, and, finally (8) disposal [11]. In line with this, the LO promotes prevention and reduction of fish waste as a top priority. However, it forfeits the second step in the hierarchy to avoid creating a market for <MRCS fish. In the case of UWC > MRCS, the LO allows for the fish to be utilised for human consumption. Since substantial efforts to create a market for these fish have not been undertaken, any landed UWC that does not have a high market value as a food product is likely to be used for animal feed production, non-food applications, or even disposed of.

At the time of writing this paper, literature on the successful utilisation of UWC in Europe was found to be scarce. This is not surprising considering that the LO was implemented only recently and the issue of UWC valorisation is rather new. DiscardLess, an EU Horizon2020 project with a focus on reducing fishing discards in the region, collated information about some ongoing initiatives. A 2019 report from the project indicated that unavoidable UWC in parts of Denmark is either discarded after landing or sold to fish meal or fish oil producers [12]. In the Netherlands and Germany, the pet food industry is the largest buyer of UWC. In Boulogne-Sur-Mer, the biggest harbour in France, UWC was largely found to be sold to a local company that specialises in the production of cosmetics, health products, and fish meal. Pescanova, the biggest fishing company in Spain, was reported to be selling UWC outside of the EU [12]. Some pilot projects focusing on valorising UWC for human consumption were found in the literature as well. Two studies from Spain showed the successful valorisation of UWC into fish mince products such as fish finger and burger patties [13,14]. A project based in northern Finland was also reported to be able to set up a fish mincing facility to utilise UWC for human consumption [15]. No literature focusing on utilising UWC for food charities in Europe could be found.

The idea of donating UWC to food banks, however, is not entirely novel. SeaShare is a US-based organisation that enables the donation of similar type of UWC to hunger relief organisations. SeaShare, as described by Watson et al. (2020), partly relies on what the US legislation calls a prohibited species donation (PSD) programme [16]. During its pilot run in 1993, the PSD program authorised the donation of salmon that was classified as prohibited species in order to prevent valuable fish protein from leaving the food supply chain [16]. Later in 1996, Amendments 26 and 29 to the Fishery Management Plans for Groundfish in the North Pacific (61 FR 38358) allowed a full-fledged programme to be established [16]. SeaShare, which is a non-profit organisation, supported the PSD programme by taking on the logistical and financial burden of processing and distributing this fish [16]. While this Prohibited Species Donation Program serves as inspiration for what could be done in the Netherlands, due to the differences in intra-industrial relationships, as well as in fishing-related laws and regulations in the US and EU, the possibilities within the Netherlands warrant a separate evaluation.

Therefore, this study explores the possibility of including more fish in Dutch food bank parcels by utilising UWC. It does this by assessing whether food bank recipients would be interested in such fish. This is followed by an analysis of relevant stakeholders’ views on valorising UWC in this manner.

## 2. Methods

### 2.1. Study Design

This mixed methods study was designed to investigate the possibility of utilising unwanted catch in Dutch food bank parcels from two perspectives. The first perspective focuses on whether food bank recipients are interested in receiving more fish in their parcels and if they are keen on consuming UWC. It was important to ascertain this because without an interest from the food bank recipients, further investigations would not be justified. Therefore, to understand whether food bank recipients want more fish in their parcels and whether they are willing to eat UWC, a quantitative approach was deemed appropriate. The goal with this approach was to survey the terrain before proceeding with further data collection. A detailed description of the method employed is provided in Section 2.2.

The second perspective looks at the fisheries supply chain and seeks to identify whether it is economically, logistically, and legally possible to utilise UWC in this manner. To answer this question, it was necessary to gather insights from stakeholders and experts working directly with this supply chain. A qualitative approach using semi-structured interviews was seen as the best fit for collecting and analysing such data since it seeks to contribute to an improved understanding of social realities [17]. It does this by drawing attention to processes, meanings, patterns, and structural features [17]. The purpose this perspective serves is to mine the surveyed terrain. A detailed description of the method employed is provided in Section 2.3.

### 2.2. Questionnaires—Statistical Analysis

Questionnaires were used to determine whether there was a demand for more fish in the food banks and if food bank recipients were willing to consume UWC. Printed questionnaires were distributed to the recipients of Food Bank Venlo during May and June 2021. Two hundred and ninety-eight paper questionnaires were packed in individual envelopes and were transported to the food bank for distribution. In the food bank, volunteers placed the envelopes in the recipients’ food parcels.

Food bank recipients had two weeks to participate in the research and involvement was entirely voluntary. The self-administered questionnaires were in Dutch and consisted of six questions, five of which were multiple-choice. The questions were asked in the following order: (1) age, (2) gender, (3) food bank visits per month, (4) frequency of receiving fish from the food bank, (5) willingness to receive more fish, and (6) willingness to eat UWC. The term UWC was explained to the participants in the introduction and repeated in question 6. The first two questions were asked to determine whether the demographic of the sample matched with that of food bank recipients from across the country. This data was also collected to examine whether age and gender were associated with desire for more fish and willingness to consume UWC. The third and fourth questions were asked for the purpose of determining whether the frequency of visiting the food bank was associated with the quantity of fish received. Finally, respondents were asked if they would be willing to eat UWC. The definition of UWC was provided along with this question but description of the fish (species, size, state) was not included. This was done in order to understand whether respondents’ interest in fish was associated with the fish’s status as UWC solely. The number of questions were limited so as to require as little of the respondents’ time as possible. The food bank recipients could either return the filled-in questionnaires during their next visit to the food bank or scan a QR-code for the online version created with Qualtrics. Returned paper questionnaires were collected from the food bank and online responses were retrieved from Qualtrics. The relationship between gender and willingness to consume UWC and age and willingness to consume UWC were tested using a chi-square test of independence.

### 2.3. Interviews

Qualitative data were collected through in-depth semi-structured interviews with experts working with the Dutch Food Bank Foundation and the fisheries sector. The theoretical sampling strategy of Glaser & Strauss (1967) was used to create a suitable sample [18]. This strategy entails collecting, coding, and analysing data simultaneously to inform decisions regarding what data to collect next and where to find them. In this approach, data collection, analysis and theory emergence take place in a parallel manner [18]. All participants were contacted via email with a brief explanation about the purpose of the study and the relevance of their participation. Ten participants were interviewed, following which recruitment was stopped because data from the interviews were no longer contributing to the further development of existing codes. This phenomenon is described by Glaser & Strauss (1967) as theoretical saturation and marks the end of data collection [18]. Table 1 provides an overview of the participants’ profiles and expertise. The interviews were conducted in English and lasted for 45 to 75 min. With the participants’ consent, interviews were recorded and transcribed *verbatim*. All participants were invited to review their interview transcripts for any inaccuracies.

As described above, the process of analysing the transcripts to identify recurring themes and ideas took place continually alongside data collection. Relevant phrases, sentences, or even entire sections were coded inductively using the in vivo technique described by Saldaña (2021) [19]. At the end of the first coding cycle, 30 codes were identified. In the next round, codes that were related to each other were grouped together into 11 categories. Subsequently, the 11 categories were further grouped together into four themes informed by the analysed data as well as previously read literature on the topic.

ATLAS.ti version 8.4 was used for all rounds of coding. The first and third authors undertook data analysis independently following the method described above and later compared and consolidated their findings. As an additional validation strategy, the last author oversaw the comparison and merging of the two independent analyses. Wherever necessary, excerpts, quoted *verbatim* unless modified to improve readability or to ensure anonymity, have been used in Section 3 to underpin the findings.

### 2.4. Ethical Considerations

Food bank recipients received the questionnaires as part of their regular food parcels. They were informed in writing about the aim of the study and that filling out and returning the questionnaires was voluntary. This approach was chosen over the researchers personally handing out questionnaires to ensure complete anonymity and so that the food bank recipients would experience no discomfort or pressure. Each envelope contained a piece of candy as acknowledgment for participation.

All participants who were interviewed were informed about the aim of the study, data storage, and privacy. Participants were given the opportunity to ask questions about these topics. They were informed that they could choose not to answer any questions asked by the researchers and to stop the interview at any given point. All participants gave their written informed consent.

## 3. Results

### 3.1. Demand and Supply

#### 3.1.1. Demand for More Fish in Food Bank Parcels

Out of the 298 distributed questionnaires, 46% were returned, and the total number of responses was 138. Gender representation was evenly split with 69 female and 68 male participants. One participant did not report their gender. The age distribution ranged from 21 to 76 years old, and the respondents’ mean age was found to be 45.23 years, with a standard deviation of 12 years. Thirteen individuals did not report their age. It was found that the study sample’s demographic closely matches that of food bank recipients throughout the country (Voedselbanken Nederland, 2020).

Out of the 138 respondents, 61 reported never receiving fish in their food parcels and 52 reported receiving fish once a month. The rest of the respondents reported receiving fish more often. However, visiting the food bank more frequently did not increase the quantity of fish received. Of the 72.46% of respondents who visited the food bank once per week, 38.00% reported that they received fish once per month. Of those who reported visiting more frequently, 37.04% reported receiving fish once per month.

Regarding food bank recipients’ willingness to eat more fish, 106 (72.45%) respondents indicated that they wanted more fish in their diet. Of these, 98.11% were willing to eat UWC. Results indicate that 81% of the female respondents wanted more fish in their diet, while 72% of the male respondents reported the same. This difference between genders with regard to desire for more fish is in line with the larger Dutch population, where women were observed to consume fish more frequently than men [20]. However, with regard to UWC, results from the chi-square test indicated that the relationship between gender and willingness to consume UWC was not significant at baseline (X^2^ (2, *N* = 136) = 4.2843, *p* = 0.117). Thus, female participants were not less or more likely than male participants to not be willing to eat UWC. Similarly, age did not show a significant association with willingness to consume UWC (X^2^ (4, *N* = 124) = 1.2278, *p* = 0.881). None of the age groups in the sample were therefore more willing to consume UWC than others. This contradicts previous studies which indicate older consumers to be more accepting of food that is traditionally seen as surplus or waste [3,21]. However, the oldest age group (50–76) is underrepresented in our sample as compared to the national food bank recipient population, and different results are possible if more individuals in the age group were to be included in the study. Table 2 and Table 3 provide an overview of gender in relation to willingness to consume UWC and age in relation to willingness to consume UWC. Missing data were not included while computing the chi-square test.

The board of the Food Bank Foundation seems to be aware of the lack of fish in the parcels. During the interviews, relevant participants acknowledged that food parcels rarely contained fish. It was mentioned that several food bank recipients could not consume various meats for religious reasons. Therefore, as per interviewees, adding more fish to the parcels would be seen as favourable by the recipients because it would improve the nutritional content of the parcels and religious or social restrictions would not apply. It was mentioned that the Food Bank Foundation was already working to identify actors in the fish supply chain who were willing to donate their surplus fish.

#### 3.1.2. Availability of Edible UWC in The Netherlands

From the results presented above, it is evident that the Food Bank Foundation and its recipients are interested in UWC. However, our inquiry regarding the availability of edible UWC did not yield as concordant a response. Neither the interviewed study participants nor existing literature could provide definitive information regarding how much edible UWC was available or discarded in the Netherlands. One of the interviewees was reliably informed that up to half of the demersal (groundfish) catch was unwanted. Other interviewees working with the fishing industry were also aware that a large quantity of fish was caught unintentionally by demersal fleets but did not provide an estimate regarding the numbers. In 2018, Stichting De Noordzee, a Dutch non-profit organisation focused on sustainability in the North Sea, reported that the Dutch demersal fisheries discarded more than 70,000 tons of fish at sea every year [22]. The Common Dab and the European Plaice were reported to be the most discarded fish [22]. The same article mentions that the LO could help reduce these numbers by stimulating the fishing industry to engineer creative ways to avoid UWC instead of landing it [22]. The article, however, does not mention what happens to this UWC when it is landed.

Based on the data collected from our interviews, it appears that UWC which is landed has three possible destinations. UWC that has demand as food for human consumption enters the food supply chain through mainstream fish auctions. UWC that does not have market value as food, including <MCRS catch that cannot be used for human consumption as per Regulation (EU) No 1380/2013, is sold for other applications such as animal feed, pet food, fish oil, food additives, pharmaceuticals, and cosmetics [23]. Lastly, unwanted catch that has no demand at all or cannot be used due to reasons such as spoilage or damage is discarded. Finding uses for all of the landed UWC is anticipated to be one of the most challenging impacts of the landing obligation [24]. There are some existing market opportunities for these fish, but as reported by Hedley et al. (2015), it is clear that new markets will need to be developed if the incoming stream of UWC is to be fully utilised [24].

If the food waste hierarchy is to be applied in this context, safe to consume UWC should stay in the human food supply chain to ensure sustainable utilisation [10]. Donating part of the UWC to the food banks could be a way to do this without creating a market for these fish. However, there are several economic barriers to this, as further discussed in Section 3.2. When asked whether it would be feasible to use UWC < MCRS in food bank parcels, none of the interviewees working with the fisheries responded enthusiastically. Some interviewees believed that if the <MCRS fish were to be utilised for human consumption, it would take away the fishing industry’s incentive to fish more selectively. Most interviewees did not think that the situation in the Netherlands was unfavourable. P10 described it as: “*The fishermen sell all their fish at the auction—target species as well as non-target species. All the fish which is marketable is being sold. There is no fish which is not being sold*”. This excludes under MCRS catch, but interviewees did not seem to view this fish as food. This can be extended to UWC without a market value in general.

Those working on the ground might have a different perspective on the issue. In a paper published by de Vos et al. (2016), all Dutch fishers interviewed for the study expressed their aversion of being obliged to land < MCRS catch due to reasons related to principle, profitability, and environmental protection [25]. An episode of the Dutch television show Keuringsdienst van Waarde that documented this issue also indicated that fishers saw this legislative requirement as unreasonable, wasteful, and unsustainable [26]. However, this does not imply that the fishers would be willing to donate this fish. As per results from Maynou et al. (2018), European fishers saw donating UWC as the least favourable valorisation route [27]. In comparison, other stakeholders like NGOs, researchers, and industry representatives expressed moderate interest. The same study compared the opinions of stakeholders by fishing regions. Stakeholders involved in North Sea fisheries considered charity to be the least favourable option [27].

In the US, a programme that facilitates the donation of UWC that cannot be used for any other purposes has been successfully established. P4, who studied the impact of this programme, emphasized the importance of stakeholder cooperation, community support, and goodwill in establishing it. Based on the responses of participants working with the Dutch fisheries, the fishing sector in the Netherlands does not seem to have any prior experience with working with food banks. As a result, cooperation or goodwill cannot be expected from stakeholders yet. NGOs or civil society organisations could play a role in brokering such an understanding between the food banks and the fishing sector. However, based on interviewees’ responses, the fisheries in the Netherlands do not currently share a cordial relationship with such organisations.

Several interviewees working with the fishing industry believed that the Food Bank Foundation should try to procure fish from a later stage of the supply chain as opposed to the pre-auction and auction stages. Frozen fish close to its expiration date, procured from various stages of the supply chain was suggested as a possible option. However, given that this study focuses on UWC, this option will not be explored further.

### 3.2. Economic Feasibility

#### 3.2.1. Paying for Unwanted Catch

Bringing UWC that is currently used for non-food applications back into the food supply chain is a sustainable way of utilising such fish. Donating it to the food bank is an attractive way to do this from the perspectives of food security and public health, but it raises concerns regarding economic viability. The food banks in the Netherlands do not pay for procuring food. Next to providing food aid, the Food Bank Foundation aims to reduce food waste by utilising surplus food that would have ended up as waste otherwise [28]. Therefore, it relies on food businesses seeing the merit of donating their surplus. In this case, however, fishers may not see the need to donate their UWC because it can be sold to other destinations and used for other purposes, such as animal feed or pet food. Income from such sale itself is unlikely to offset the costs incurred from keeping UWC on board [29]. However, donating UWC instead would further reduce profits. Interviewees working with the fishing industry expressed their understanding regarding donation being the more sustainable option but also regarded it as an unlikely scenario due to its impact on profits. Considering that implementing the LO is likely to cost demersal cutter fleets between EUR 5.6 and 12.3 million in transition costs [29], the sector’s worries regarding profits and economic viability are well founded.

Additionally, the market value of UWC is dynamic. One of the interviewees described the example of the octopus to demonstrate this: “*Five years ago, octopus was a low-value side catch. It was sold at 2 euros per kilogram. But these days, it has a value of around 18 euros per kilogram. Nowadays, we see that the demand for fish is healthy and even the side-catch is expensive*”. In hopes of receiving a higher price for their UWC in the future, fishers may not want to donate their UWC to the food bank and classify it as low value fish.

When asked whether paying a small amount of money for procuring UWC was something the Food Bank Foundation would consider, interviewees working with the food banks conveyed that this would not be feasible. Interviewees indicated that over the last years, the food banks’ supply has decreased while the number of people signing up to receive food aid has increased. Additionally, they believed that if the foundation started paying for one category of products, all their procurement partners would expect to be paid.

#### 3.2.2. Processing Costs

Economic barriers to donating UWC do not end at the procurement stage. If we were to consider the hypothetical scenario of fishers being willing to donate their low value UWC to the food banks, processing costs would still be a concern. Processing the fish would be necessary because providing whole, unprocessed fish to the food bank recipients might lead to them wasting it as a result of not knowing how to handle it. A cross-cultural study by Olsen et al. (2007) found the Dutch population to have the least positive outlook towards preparing fish at home compared to the other four European countries considered in the sample [30]. Given that the food bank recipients are known to consume particularly low quantities of fish [7], interviewees working with the food bank agreed that it would be important to introduce UWC in an accessible manner. Therefore, processing costs must be taken into consideration. This challenge might be easier to overcome than paying for procuring fish. Although the Food Bank Foundation does not spend on procuring food, interviewees stated that it is willing to do so for setting up infrastructure to process the food that it procures. Currently, food banks source most of their products from the retail stage of the supply chain. However, P1 mentioned that the board was looking to procure at least a part of its supply from earlier stages of the supply chain because the retail sector was becoming increasingly efficient at managing its surplus. Procuring from earlier stages of the supply chain would entail some degree of processing and as per the information provided by relevant participants, the board of the Food Bank Foundation is looking into the possibility of creating a separate foundation that handles the procurement and processing of such food. However, processes such as filleting fish are highly specialised and relatively expensive. It is therefore important to undertake a thorough cost-benefit analysis before investing in such facilities. It is important to note, however, that utilising fishing by-products is not completely new to the fishing industry. Operations to valorise fish by-products including cutting, boiling, drying, and ensiling exist across European harbours but limited attempts to introduce UWC in existing manufacturing operations have been made [11,27].

If UWC < MCRS is to be utilised in food bank parcels, processing costs might be an important factor to consider given the small size and bony structure of such fish. However, interviewees working with the Dutch fishing industry were far less willing to discuss the possibility of donating under MCRS catch to the food banks. Some interviewees mentioned that utilising < MCRS catch in other sustainable ways was not economically viable either. Regarding such trials, they elaborated: “*Fishers are not allowed to sell certain unwanted catch for human consumption, so it must go for products like pet food, fish meal, and fish oil. For demersal species, this is just not economically viable. We did look at whether you could extract high level proteins or turn it into fish oil instead of using it for pet food for instance. But the problem is that they would have to treat the unwanted catch in accordance with the same quality standards they use for their commercial fish. That is impossible*”.

Upon enquiring if they considered this fish to be safe for consumption, all answered favourably. However, they stated the LO requirement to not use this fish for human consumption as the restricting factor. When we enquired about the same issue but in a hypothetical situation where the LO allowed the donation of UWC < MCRS to the food bank, interviewees began to propose other profitable ways of utilising this fish. Another hypothetical scenario suggested by the researchers focused on the government providing monetary compensation to fishers for donating UWC or UWC < MCRS to the food banks. This option was not seen as favourable due to the possibility of it turning into a perverse incentive. However, targeted benefits such as tax deductions or subsidies related to sustainability were viewed more favourably. In the US context, P4 described similar incentives to have worked positively for the fish donation programme.

#### 3.2.3. Competition from Non-Food and Black Markets

UWC that does not have a high market value as food is redirected to animal feed, pet food, or other technical uses. These supply chains are well established in the Netherlands and would therefore pose as a barrier to entry for the food bank. Some interviewee responses indicated that selling UWC for non-food applications is not favoured by the fishers, but given that they are able to recover a part of their costs through these transactions, they cannot refuse them. Despite not bringing in as much value as the food supply chain, non-food chains are important to the fishing industry. For instance, several participants mentioned that the recent shutting down of Dutch mink farms had negatively impacted the fishing industry because mink farmers no longer purchased fish as food for their minks. One participant expressed the situation as follows: “*Feed producers pay maybe seven to eight cents per kilo of this fish and that is not the best way to bring these proteins back into the chain. Seven cents do not make the fishers happy anyway. One cent more than the feed producers and they would rather give it to the food bank*”. However, as discussed above, the Food Bank Foundation is unwilling to pay for procuring this fish. Selling UWC to fish meal and oil producers is also seen as more advantageous than donating it to charity because these well-established supply chains are known to be able to cope with uncertainties like fluctuations in composition and quality [27]. Fish meal is also likely to be seen as an economically advantageous choice because Europe is a net importer of fish for non-food use [31].

Some interviewees mentioned that the existence of black markets for under MCRS catch might pose as a barrier for donating it to the food banks. It was suggested that legalising the use of UWC < MCRS in food banks may help these shadow markets proliferate and make it more challenging for national authorities to implement the LO. One of the participants stated, “*if a fishing vessel is found with undersized fish being stored in a frozen state for sale as food, the fisherman could simply say that it is all for the food bank and then sell it illegally*”. Bellido et al. (2017) discuss this in the context of the Mediterranean region wherein they note that implementing the LO may lead to the expansion of illegal markets for fish below the minimum size [32]. However, this is discussed in the context of simply keeping the fish onboard to land it and not donating it to food banks.

### 3.3. Logistics and Infrastructure

#### 3.3.1. Fishing Industry’s Perspective

Prior to the LO being implemented, fishers were able to select what fish to keep on board and what to discard or release at sea. As a result, fishing vessels have been designed to accommodate catch that can be brought ashore and sold at an attractive price [33]. Interviewees mentioned that vessel infrastructure and labour costs would be a cause for concern if fishers were expected to treat UWC in the same way they did regular catch. If UWC is to be used by the food banks, a high level of food safety will have to be ensured by applying standard bleeding, cleaning, sorting, and cold storage procedures. This, in turn, will require labour, space, and machine capacity on board. Without any monetary returns, fishers are unlikely to be willing to undertake these tasks. Even in the current situation where UWC can be sold at a low price for non-food purposes, fishers are not in favour of processing and accommodating this fish onboard [33].

When it comes to infrastructure related investments, interviewees working with the fishing industry in the Netherlands stated that the sector is heavily focused on developing fishing gear that will enable more selective fishing, thereby eliminating UWC. Based on interviewee responses, developing vessel capacity to handle UWC can be seen as a contradiction to improving fishing gear and, as a result, fishers might be unwilling to invest in it. The work of Viðarsson et al. (2019) confirms that vessel owners are reluctant to invest in technology to process and preserve UWC onboard [33]. The same paper suggests silage production from <MCRS catches onboard the vessels [33]. This silage could be used for producing fish meal for animal feed and other uses but render it unusable for food bank use.

It was not possible to access information regarding how UWC is currently handled aboard Dutch vessels. Generally, the main challenges for onboard management are associated with catches under MCRS [33]. UWC > MCRS is often destined for human consumption and can therefore be managed as per the traditional onboard handling processes.

#### 3.3.2. Food Donation Logistics

If we were to assume that the fishing industry is willing to cooperate with the food banks for the donation of UWC < MCRS, the Food Bank Foundation would need to invest in suitable logistics and infrastructure to be able to use this fish. Although fish is rarely provided in food bank parcels, the cold chain for frozen meat products is already established. As per relevant interviewees, the Food Bank Foundation would need to expand its current cold storage and transportation facilities if it was to receive more fish. This investment was seen as viable if a regular flow of processed, ready-to-cook fish could be ensured. However, given that the fishing industry’s objective is to fish more selectively and reduce UWC, ensuring a steady flow of fish for the food bank could be challenging. Considering these uncertainties, it would be seen as risky for the Food Bank Foundation to invest in expanding its cold chain specifically for fish. A possible solution to this could be renting such logistics facilities. As per the information provided by interviewees, the Food Bank Foundation already rents such a warehouse to manage its current flow of frozen products.

Regarding food safety, relevant interviewees were confident about the volunteers being able to handle fish if the food banks were to receive it processed and then frozen. The food banks work with an in-house private standard for food safety which already includes provisions for handling fish. Therefore, it can be expected that ensuring the safety of UWC in food bank parcels is feasible if it is handed over to the food banks in good condition.

Dutch food banks are likely to be able to incorporate more fish in its inventory more easily compared to the US fish donation programme described by P4 and P5. The programme described by them is based in Alaska and required significant infrastructural investment to make it operational. The Dutch food banks, in comparison, have a well-established system for procuring, transporting, storing, and redistributing surplus food, including products that need cold storge facilities. However, based on interviewee responses, the fishing industry may not feel confident about food banks’ capacity to handle UWC. One of the interviewees described that the industry itself engages in charitable acts but the food banks may not be able to replicate this: “*Sometimes fishers donate fish for social causes. One or two times a year, they participate in a project for homeless people in Rotterdam where they donate fish, fry it, and give it to the homeless people. But if you want to incorporate fresh fish into the parcels of the food banks, that’s going to cause a bit of a logistical problem because the food banks are absolutely not equipped to handle whole fish that is not processed*”.

### 3.4. Fisheries Policy and Legislation

The Common Fisheries Policy and the Landing Obligation and their impact on UWC valorisation came up as a prominent theme in our analysis. The LO, and by extension the CFP, share a rather paradoxical relationship with the research question this study seeks to answer. The possibility of donating UWC to the food banks arises largely because fishers are now obliged to land fish that they unintentionally catch. Prior to the implementation of Regulation (EU) No 1380/2013, fishers could simply discard UWC at sea. It is only due to the Landing Obligation that this fish will need to be brought ashore, thus giving rise to surplus fish that needs to be utilised. At the same time, the LO also creates barriers for donating this fish to the food banks.

Firstly, the fishing industry’s discontentment towards the LO may negatively impact its willingness to donate UWC. One of the interviewees working in close cooperation with the fishers described the LO as following: “*This landing obligation is not workable. It’s not doable. It’s not enforceable. We need to turn away from this non-workable regulation and move towards a workable regulation. But everyone is so politicised. And everybody is so concerned with the issue of discarding at sea. Nobody on the policy side wants to give in*”. This sentiment of the fishing industry towards the LO has been discussed in existing literature as well [25,34,35,36]. To prepare for the implementation of the LO, the Dutch government launched a working group (werkgroep Aanlandplicht) in autumn 2012 [36]. Scientists, NGOs, industry representatives, and civil servants from the ministry and the control agency were invited to be part of this working group [35]. However, the Dutch fishing industry reacted negatively to the possibility of having to land UWC and refused to participate in the working group [35]. Based on interviewee responses, it is evident that the fishing industry continues to hope that legislators will take the fishers’ dissatisfaction into consideration and repeal the requirement to land UWC. Establishing a system where UWC is donated to the food banks might create dependency on landing UWC and therefore make it difficult to reverse LO. Regarding the push to make amends to the LO, an interviewee working on fisheries governance in the Netherlands elaborated: “*There is a strong call to re-evaluate the landing obligation. Everybody in the industry says it’s not working. They say it’s actually contributing more to illegal fisheries. The fishers are so worried about fisheries being closed and therefore, about their survival. We are very lucky to still be allowed to go on board and see the actual catch composition for research. But we also know from colleagues in other countries that fishermen tell them: ‘Sorry, we cannot take you anymore, because you would see that we are actually discarding unwanted fish at sea’*”.

Secondly, the restriction on using UWC < MCRS for human consumption impedes the possibility to donate this fish. Interviewees frequently stated that donating < MCRS fish to the food banks would not be possible because (Article 15(11) of) Regulation (EU) No 1380/2013 does not permit the use of such fish for human consumption [23]. They viewed this requirement as non-negotiable and central to the LO. However, during the formative stages of this legislation, the sale of UWC < MCRS for direct human consumption was hotly debated [37]. Some Member States, especially those from the Baltic basin, proposed that once the undersized fish are caught and counted against quota, they should be given appropriate value [37]. They expected that this value would be low enough to deter fishers from catching too many undersized fish [37]. However, other Member States, particularly from the Mediterranean basin, were of the opinion that this could encourage fishers to catch undersized fish because, unlike the Baltic, consumer demand for small fish is high in the Mediterranean region [37]. As a compromise, legislators concluded that UWC < MCRS should be landed, counted against quota, and then sold only for non-human consumption [37]. These diverging views on the use of <MCRS coupled with the fact that consumer demand for small fish varies significantly across the continent may indicate that one policy may not fit all Member States. It is important to note that the decision to not allow the direct human consumption of <MCRS was based on general knowledge and is not supported by empirical evidence [37].

This reluctance to consider that undersized fish could be used for human consumption can be seen in other pieces of European legislation too. For instance, the 2013 proposal to regulate fishing in the Skagerrak reflects this [38]. In the draft version, Article 5 initially stipulated the following: *‘(…) the sale of catches of that stock below the minimum conservation reference size shall be restricted to reduction to fish meal, pet food or other non-human consumption products only, or for charitable purposes.’* The final version of the proposal omitted ‘*or for charitable purposes’*. A report recording the decision-making process explained that this was done because *‘it is not appropriate that juveniles be sold for charitable purposes’* [39]. Further, it stated ‘*it would have been possible to amend the provision so that juvenile fish may be given to charitable purposes, but as there is no such tradition in the Member States surrounding the Skagerrak, such a provision would have no place in the Regulation’* [39]. This goes on to show that the aversion to donate UWC for use as food or for charity is based on arbitrary factors rather than scientific evidence.

## 4. Discussion

The aim of this research was to explore the possibility of including more fish in Dutch food bank parcels by utilising unwanted catch. This study is socially and environmentally relevant because donating UWC to the food banks could not only improve the nutritional quality of the food parcels but also reduce food waste from the fishing industry. Based on the results discussed above, economic and legislative barriers, stakeholder relations, and the state of logistics and infrastructure would currently make the donation of UWC to food banks challenging. In this section, we discuss the possibility of overcoming these barriers.

The push to ban discards at sea came from a place of concern regarding food waste. Discussions about high volumes of discards in European fisheries were ongoing at the Commission when British celebrity chef Hugh Fearnley-Whittingstall’s 2010 public campaign gained popularity across the Member States. Celebrities, influential retailers, environmental NGOs, and the general public expressed solidarity with Fearnley-Whittingstall’s demand to end the wasteful practice of discarding UWC at sea [36]. The campaign, backed by over 650,000 petitioners, played an important role in influencing legislators to pass a law requiring fishers to land their unwanted catch instead of discarding it at sea [25,34,36]. The Commission hoped that the LO would encourage the fishing industry to focus its attention on developing selective fishing practices and eventually, significantly reducing the existence of UWC. However, developing selective fishing techniques that reduce discard rates to the 5% benchmark set by the CFP cannot be realised instantaneously. This is especially true for fisheries such as the demersal fleets that fish in the North Sea where the discard rate has historically been as high as 40% [40]. Until such tools and technologies are developed and successfully implemented, UWC will continue to be landed. The initial emphasis on food waste that led to the realisation of LO was eventually replaced by concerns regarding the health of fish stocks and marine ecology. Neither the CFP nor connected policy areas provide Member States with guidelines regarding how surplus fish that would be landed as a consequence of the LO should be sustainably utilised. The policy formulation process can be considered top-down due to the fishing industry’s minimal involvement in it. The fishing sector’s aversion to this approach is evident in the results. De Vos et al. (2016) indicate that fishers feel that their professional knowledge was disregarded by policymakers and scientists who lack a pragmatic understanding of how the industry is organised [25]. In their paper on the US-based seafood donation programme SeaShare, Watson et al. (2020) describe that a flexible, bottom-up approach coupled with regulatory changes enabled the programme’s success [16].

In the European context, literature recognising the food waste problem associated with the Landing Obligation is scant. The European Court of Auditors’ 2016 report on food waste is one of the few public documents that acknowledges that UWC landed as a result of the LO could end up as food waste [41]. The report describes the lack of legal provision to donate UWC in the new CFP as a missed opportunity. Furthermore, it encourages the Commission to assess the possibility of including legal provisions in the CFP to donate surplus fish [41]. The paper published by Vaqué (2017) makes an identical appeal [42]. The EU FUSIONS research project (2012–2016) also identified the risk of the LO turning fish waste at the sea into food waste on land [43]. This paper adds to the limited body of literature focusing on this issue and recommends better cohesion between the CFP and the EU’s food waste reduction ambitions. Including a legal provision to donate surplus UWC would be a first step in facilitating this. It is unlikely that such provisions would discourage selective fishing. Landing unwanted catch would still be seen as unfavourable by fishers due to limited onboard storage and low value fish getting counted against quota. The incentive to fish selectively would considerably outweigh the incentive to land UWC and donate it to charity.

If donations are to be operationalised in the EU, legal provisions will have to be supported by suitable infrastructure. One way to facilitate this could be through the European Maritime Fisheries and Aquaculture Fund (EMFAF) which entered into force in July 2021. Part of its EUR 6,108,000,000 budget is allocated to projects focused on ensuring food security through the supply of seafood products in the Member States [44]. Additionally, the fund was set up to help fulfil the objectives of the EU Green Deal, which explicitly states reducing food waste as one of its goals [45,46]. Donating UWC to food banks not only improves food security through seafood products but also reduces food waste and ensures that UWC is utilised sustainably. To safeguard fishers’ economic interests, such projects could be supported by public-private partnerships. Iñarra et al. (2020) show that turning UWC > MRCS into fish burgers or other minced fish derived products is an economically viable way to utilise UWC while also increasing fish consumption [14]. If the fish is delivered to consumers, including food bank recipients, in an affordable and easy to cook format, it is likely to be well received. The paper published by Iñarra et al. (2020) describes results from one of the few case studies that explore the possibility of utilising UWC for human consumption and will need to be replicated in different contexts. However, it considers UWC < MRCS to be ineligible for such operations largely due to the legal restriction on using such fish for direct consumption [14]. If the EMFAF is utilised to set up operations to produce new fish products from UWC and if direct human consumption of UWC < MRCS is permitted for charitable purposes, part of the fish products can be donated to the food banks. This could help ease the fishing industry’s concerns regarding the economic consequences of donating UWC. At the same time, such funding and state support could help the food banks set up infrastructure and logistics to safely handle the fish.

Voluntary agreements as a form of private governance could also help advance the sustainable utilisation of unwanted catch. EU REFRESH (2015–2019), a project focused on reducing food waste across the EU, recommended voluntary agreements (VAs) as a tool for private actors to fill legislative gaps with regard to food waste valorisation [47]. VAs can be used as an alternative course of action to traditional legislation and can be directed by government officials, businesses, or other actors [47]. In the context of UWC utilisation, the VA could focus on developing new markets, setting up infrastructure, and discouraging the illegal trade of UWC. Donating a share of the UWC to improve food security could be a part of the agreement. Relevant actors who could collaborate include fishing industry associations, fish processors, NGOs, and the Food Bank Foundation. The EMFAF or other national funds set up for the purpose of improving food security could help finance such programmes.

Some limitations faced by existing food waste-focussed VAs in the Netherlands are already known. It is important to take these into account while developing any new agreements on UWC valorisation. For instance, Piras et al. (2018) point out that besides SDG 12.3, the Netherlands does not have a specific national food waste reduction target. Due to the lack of dedicated policy measures, government support for voluntary actions is limited [47]. The same report also indicated that a paucity of food waste data makes it challenging to arrive at objective targets. Current food waste VAs in the Netherlands also lack built-in sanctions for non-compliance [47]. This makes it possible for free riders to take undue advantage of such agreements by joining them only to improve their public image without taking concrete action to reduce food waste [47]. Next to this, prior to setting up any programmes, taking demand-side barriers into consideration is critical. To address such barriers, van Putten et al. (2019) recommend selling unwanted catch at an affordable price and educating consumers about preparing such fish [48]. Efforts to increase consumer acceptance should be directed towards the general public and not only food bank recipients because several types of UWC can be used for direct human consumption.

### Strengths and Limitations

This paper makes a first attempt to analyse whether unwanted catch that is landed as a consequence of the new EU Common Fisheries Policy can be utilised to improve food security. It adds valuable insight to a limited body of literature that discusses the issue of unwanted catch through the lens of food waste valorisation. It is, however, not without limitations. Not all relevant stakeholders could be interviewed for this research. Some stakeholders (*n* = 7) invited to take part in this study did not agree to participate. Additionally, fishers, who would be the ultimate decision makers regarding the donation of UWC, were not included in the sample. This is due to the scope of our enquiry being limited to understanding this previously unexplored issue from a bird’s-eye view as opposed to mapping the reality on the ground. Future research on this topic could highlight the viewpoint of legislators, policymakers, and fishers, thereby filling the gap in the literature. Next to this, despite employing validation strategies such as peer reviews and multi-author coding while analysing data from the interviews, a certain degree of bias could be present in this study due to its qualitative nature.

Lastly, quantitative data was collected by recruiting participants from only one out of the 171 food banks in the Netherlands. Whether food bank recipients across the country share the same inclination towards eating UWC remains unknown. The respondents were only provided with the definition of unwanted catch. Additional information such as fish species and condition (processed, unprocessed), and taste tests might yield different results. However, prior to this research, it was not known whether the food banks and their recipients were inclined to receive more fish. Their attitude concerning unwanted catch had never been studied either. Results presented in this study open the doors to further enquiry on this issue.

## 5. Conclusions

This study explored the possibility of including more fish in Dutch food bank parcels by utilising unwanted catch. It did this, firstly, by gauging whether food bank recipients would be interested in such fish. This was followed by an analysis of relevant stakeholders’ opinions on such an initiative. By considering unwanted catch utilisation, fish shortage in Dutch food bank parcels, and food waste valorisation together, this paper paves the way for a better understanding of all three issues and their possible interconnectedness.

Currently, several economic, legislative, social, and logistical barriers stand in the way of donating unwanted catch to food banks. However, given the European Commission’s, and in turn the Dutch government’s, strong focus on achieving the SDGs, change is possible. As highlighted in this paper, sustainably utilising unwanted catch and reducing food waste are often not viewed by policymakers as interconnected issues. Through this study, we aim to challenge this narrative. Legislative change, sufficient funding, and industry-led initiatives such as voluntary agreements can make donating safe-to-consume surplus fish to food banks a reality in the near future. Such an initiative could potentially improve health, accommodate the increasing demand for sustainable protein, and prevent wastage of valuable marine resources.

## Figures and Tables

**Table 1:** Overview of participants and their expertise.

Participant	Expertise	Description
P1	Supply chain	Procurement and supply chain specialist connected with the Netherlands Foodbank Association.
P2	Market research	Market researcher connected with the Netherlands Foodbank Association.
P3	Food safety	Food safety specialist connected with the Netherlands Foodbank Association.
P4	Marine ecology	Research scientist based in the US, specialising in quantitative marine ecology.
P5	Marine ecology	Research scientist based in the US, specialising in marine conservation ecology.
P6	Fisheries governance	Independent consultant having extensive experience with NGOs focused on sustainable fishing.
P7	Fisheries governance	Project manager for sustainable fisheries governance programmes in the Netherlands.
P8	Fish trading and processing	Commercial head for a fish trading and processing business in the Netherlands.
P9	Fish trading and management	Director for a national fish auction in the Netherlands.
P10	Fisheries political affairs	Director for various association advocating for the interests of fishers in the Netherlands.

**Table 2:** Gender in relation to the willingness to consume UWC.

	Total Number of Participants (% by Gender)	Willing * (% m/f)	Not Willing * (% m/f)	Unsure * (%m/f)	Missing Values	*p*-Value
Gender						0.117
Male	68 (49.3%)	52 (47.7%)	10 (76.9%)	6 (42.9%)		
Female	69 (50.0%)	57 (52.3%)	3 (23.1%)	8 (57.1%)	1	
Missing	1	1	0	0		

* = Reported willingness to consume UWC.

**Table 3:** Age groups in relation to the willingness to consume UWC.

	Total Number of Participants (% by Age Group)	Willing * (% by Age Groups)	Not Willing * (% by Age Groups)	Unsure * (% by Age Groups)	Missing Values	*p*-Value
Age group						0.882
18–29	15 (10.9%)	12 (12.1%)	2 (16.7%)	1 (7.1%)		
30–49	67 (48.5%)	53 (53.5%)	7 (58.3%)	7 (50.0%)		
50–76	43 (31.2%)	34 (34.3%)	3 (25.0%)	6 (42.9%)		
Missing	13	11	1	0	1	

* = Reported willingness to consume UWC.

## Data Availability

The corresponding author can be contacted to request a copy of the code book.
